# Tooth-Whitening Agents and Polymer-Based Carriers: Efficacy, Safety, and Clinical Perspectives

**DOI:** 10.3390/polym17182545

**Published:** 2025-09-20

**Authors:** Pin-Yu Lin, Li-Nai Chen, Chien-Fu Tseng, Yi-Shao Chen, Hung-Yu Lin, Thi Thuy Tien Vo, Tzu-Yu Peng, I-Ta Lee

**Affiliations:** 1 School of Dentistry, College of Oral Medicine, Taipei Medical University, Taipei 11031, Taiwan; b202113017@tmu.edu.tw (P.-Y.L.); b202113033@tmu.edu.tw (Y.-S.C.); b202113010@tmu.edu.tw (H.-Y.L.); 2 Department of Dentistry, Taoyuan General Hospital, Ministry of Health and Welfare, Taoyuan 33004, Taiwan; blacky09@tmu.edu.tw (L.-N.C.); d02422002@ntu.edu.tw (C.-F.T.); 3 Research Center for Tooth Bank and Dental Stem Cell Technology, College of Oral Medicine, Taipei Medical University, Taipei 11031, Taiwan; 4 Graduate Institute of Clinical Dentistry, School of Dentistry, College of Medicine, National Taiwan University, Taipei 10048, Taiwan; 5 Faculty of Dentistry, Nguyen Tat Thanh University, Ho Chi Minh City 700000, Vietnam; vtttien@ntt.edu.vn

**Keywords:** tooth whitening, whitening agents, hydrogen peroxide, polymer-based formulations, biocompatibility

## Abstract

Tooth whitening is increasingly sought in both clinical and home settings, raising concerns about the efficacy and safety of various whitening agents and their delivery systems. This narrative review compares the whitening performance and biocompatibility of active ingredients, including hydrogen peroxide, carbamide peroxide, activated charcoal, sodium bicarbonate, fluoride compounds, and blue covarine, with particular emphasis on the role of polymer-based carriers in formulation strategies. Hydrogen peroxide and carbamide peroxide remain the most effective agents for intrinsic whitening, but are associated with risks of enamel surface alterations, microhardness reduction, and potential cytotoxicity, particularly at higher concentrations. Sodium bicarbonate provides moderate whitening effects through extrinsic stain removal, while fluoride compounds play a supportive role by reducing demineralization and tooth sensitivity, thereby preserving enamel integrity. These properties make them valuable adjuncts or alternatives for patients with high sensitivity risks. Blue covarine offers immediate optical effects without inducing intrinsic color changes, whereas activated charcoal poses risks of enamel abrasion and surface roughness with limited long-term efficacy. Polymer-based carriers such as Carbopol gels, polyvinylpyrrolidone, and hydroxypropyl methylcellulose are incorporated into whitening formulations to improve viscosity, adhesion, and modulate the release of active ingredients. These polymers might help minimize diffusion of bleaching agents into deeper dental tissues, potentially reducing cytotoxic effects, and may improve handling characteristics. However, dedicated studies evaluating the unique advantages of polymers in different whitening systems remain limited. A comprehensive understanding of both the active ingredients and delivery technologies is critical to balancing esthetic outcomes with long-term oral health. From a clinical perspective, polymer-based carriers might contribute to reducing whitening-related tooth sensitivity, improving patient comfort, and providing more predictable treatment outcomes. Continued research is needed to clarify optimal formulations and application protocols, ensuring safer and more effective tooth-whitening practices in both clinical and home-use scenarios.

## 1. Introduction

Tooth whitening has become one of the most prominent esthetic procedures in modern dentistry, with professional treatments demonstrating high efficacy alongside the widespread use of at-home methods to enhance dental appearance [1]. Common whitening agents include hydrogen peroxide, which has been clinically validated at varying concentrations for in-office bleaching [2]; carbamide peroxide, whose effectiveness is concentration dependent as shown in in vitro studies [3]; and abrasive materials such as activated charcoal, which provide measurable whitening effects but raise concerns regarding enamel abrasion [4]. These agents differ in their mechanisms of action, whitening efficacy, and duration of effect. Despite their benefits, whitening agents raise important safety concerns. High concentrations of bleaching gels can compromise enamel microhardness and alter surface morphology [5], while carbamide peroxide has been reported to induce cytotoxic effects on odontoblast-like cells [6]. Moreover, comparative in vitro studies indicate that even over-the-counter (OTC) products may pose risks to enamel integrity and biocompatibility when compared with hydrogen peroxide [7]. These findings highlight the need to balance whitening efficacy with long-term safety. Clinicians must carefully consider both esthetic outcomes and preservation of oral tissues, as inappropriate use of whitening products may lead to enamel demineralization, heightened sensitivity, or pulp irritation [5,8]. In particular, OTC products such as activated charcoal-based formulations may provide whitening benefits but also exhibit notable abrasiveness, underscoring the importance of patient education regarding ingredient awareness and safe application [4]. Driven by consumer expectations for improved appearance and procedural safety, the global tooth-whitening market has grown rapidly [1,7]. Beyond the active whitening agents themselves, recent developments emphasize the critical role of delivery systems in determining both efficacy and safety [2,3,5,8]. Polymer-based carriers have become increasingly integral to whitening formulations. For example, polyvinyl acetate (PVAc), ethyl cellulose (EC), and polyvinylpyrrolidone (PVP) have been incorporated into hydrogen peroxide gels; PVAc and EC improve adhesion and whitening efficacy, while PVP enhances stability through interactions with peroxide [9]. PVP is also recognized for its biocompatibility, solubility, and capacity to improve controlled release in oral drug-delivery systems [10]. Such polymers not only increase viscosity and adhesion to tooth surfaces but also directly interact with whitening agents at the molecular level. PVP, for instance, stabilizes hydrogen peroxide through hydrogen bonding, reducing premature decomposition, while Carbopol’s cross-linked structure functions as a gel matrix that modulates peroxide diffusion, which may be relevant for controlling its penetration in dental applications [9,10]. Hydroxypropyl methylcellulose (HPMC) is widely applied in controlled-release formulations because of its thickening, gelling, and swelling properties, enabling stable hydrogel formation and sustained release of active agents [11]. Its role as a functional excipient in hydrophilic matrix systems has been well recognized for supporting controlled release of active compounds [12], which may in turn be relevant for modulating peroxide delivery in whitening formulations. Additionally, polymer-based matrices allow the incorporation of protective additives such as calcium compounds and fluoride, which synergistically promote remineralization and reduce mineral loss during bleaching [13,14,15]. Both clinical and in vitro studies provide distinct yet complementary insights into the performance and safety of whitening agents and delivery systems. Clinical trials assess treatment efficacy and patient outcomes under real-world conditions [2,8], whereas in vitro studies enable mechanistic evaluation under controlled conditions, including enamel demineralization, oxidative stress, and cellular toxicity [5,6,7]. However, in vitro models cannot fully replicate the dynamic oral environment [4,7], while clinical trials are often limited by sample size and participant variability [1,2,8]. Integrating findings from both approaches is therefore critical for evidence-based clinical decision-making. This review aims to comprehensively compare commonly used tooth whitening agents and their biocompatibility, with a particular focus on the molecular interactions between whitening agents and polymer-based carriers. Understanding these interactions may inform the design of safer, more effective, and patient-centered whitening therapies. Clinically, polymer-based systems have the potential to reduce whitening-related sensitivity, improve patient comfort, and deliver more predictable outcomes. By combining insights from both clinical and laboratory research, this paper seeks to provide guidance for clinicians and patients in selecting safe and effective whitening strategies.

## 2. Tooth-Whitening Agents and Delivery Systems

### 2.1. Hydrogen Peroxide

#### 2.1.1. Whitening Efficacy and Color Evaluation

Hydrogen peroxide is among the most widely used and effective agents for tooth whitening. Its mechanism of action involves the generation of reactive oxygen species (ROS) that oxidize pigmented compounds within dental hard tissues, leading to visible color improvement. The clinical efficacy of hydrogen peroxide has been consistently supported by both randomized controlled trials and laboratory investigations [2,16]. Across various concentrations, it demonstrates substantial whitening potential, although the relationship between concentration and efficacy is not strictly linear [2,5]. In a double-blinded randomized controlled trial with 54 participants, Altınışık et al. [2] compared in-office bleaching protocols using hydrogen peroxide at concentrations of 18%, 25%, and 40%. All groups achieved significant improvements in tooth color, as measured by both ΔEab and ΔE00 values. Notably, higher concentrations did not consistently produce superior outcomes beyond the initial treatment effect. Instead, additional whitening was observed with 18% and 25% hydrogen peroxide after a second application, suggesting that protocol optimization, such as adjusting treatment frequency or application time, may be more clinically relevant than merely increasing peroxide concentration [2].

#### 2.1.2. Biocompatibility and Safety Considerations

Despite its proven efficacy, hydrogen peroxide raises concerns regarding enamel integrity and cytotoxicity, particularly at higher concentrations [5,6]. Melo et al. [5] reported that bleaching gels containing 35% hydrogen peroxide induced pronounced enamel surface irregularities and an approximate 18.3% reduction in microhardness, indicating an increased risk of demineralization. To mitigate such adverse effects, a variety of adjunctive strategies have been investigated. Fernandes et al. [13] showed that calcium-substituted sodium trimetaphosphate (CaNaTMP) can reduce mineral loss and peroxide diffusion, preserving enamel integrity during bleaching. Other studies have further suggested potential benefits from bioactive glass, self-assembling peptides, and natural antioxidants such as quercetin, which may restore or maintain enamel microhardness without reducing whitening efficacy [16,17]. In addition, nano-hydroxyapatite (n-HAp) has been reported to improve light reflection, enhance perceived brightness, and reduce mineral loss, supporting its role as an effective adjunct in whitening treatments [18,19]. These findings highlight the importance of concentration control and the incorporation of both polymeric and non-polymeric additives to improve the safety profile of hydrogen peroxide gels [13,14,15,16,17,18,19].

#### 2.1.3. Role of Polymer-Based Carriers

Delivery systems are fundamental to the formulation and clinical application of hydrogen peroxide bleaching agents. Many commercial products are designed as gel-based systems, which allow precise placement on dental surfaces and reduce inadvertent exposure to adjacent soft tissues [2,5]. However, high-concentration hydrogen peroxide gels have been linked to adverse effects on enamel, including increased surface roughness and reduced microhardness, underscoring the need for mitigation strategies [5]. Polymers incorporated into bleaching formulations not only serve as carriers but also play active roles in stabilizing peroxide and modulating its diffusion. PVP, for instance, interacts with hydrogen peroxide via hydrogen bonding, as demonstrated in computational studies. Its established use as a pharmaceutical excipient supports its stabilizing effect, which prolongs peroxide activity during application [9,10,20]. This stabilization is attributed to the carbonyl group of the PVP lactam ring interacting with the hydroxyl groups of hydrogen peroxide, thereby reducing spontaneous decomposition [20]. Carbopol, a cross-linked polyacrylic acid derivative, creates a highly viscous hydrogel matrix that functions as a diffusion barrier, thereby modulating the release and penetration of incorporated small molecules [21,22], a property that may be relevant for controlling peroxide behavior in dental formulations. Its abundant carboxyl groups (–COOH) ionize in aqueous environments to form an anionic network capable of entrapping small molecules and restricting diffusion [21,22]. Although direct evidence for peroxide entrapment is limited, this mechanism may help explain the reduced cytotoxicity observed in certain Carbopol-based formulations [6,15]. Further studies are required to directly confirm this mechanism in bleaching gels. HPMC enhances gel viscosity and modulates release kinetics, enabling a more gradual and sustained delivery of active agents [11,12]. While its controlled-release properties are well established in pharmaceutical formulations, further validation is needed to confirm their relevance in bleaching applications. Despite the widespread application of polymer-based carriers in whitening products, further studies are needed to clarify their precise contributions to peroxide stabilization, diffusion control, and oxidative modulation [5,13,14]. Other polymers frequently employed in commercial bleaching or oral care formulations include ammonium acryloyldimethyltaurate/VP copolymer (e.g., Aristoflex^®^ AVC), PVM/MA copolymer, hydroxyethylcellulose, and xanthan gum, which are valued for their rheology-modulating, film-forming, and sustained-release properties [23,24,25]. The integration of advanced polymer delivery systems with adjunctive protective agents, including non-polymeric remineralizing additives, represents a key strategy for optimizing both the safety and therapeutic efficacy of hydrogen peroxide-based whitening interventions [9,13,14,15].

#### 2.1.4. Adjunctive Protective Additives (Non-Polymeric)

Beyond polymer-based carriers, several non-polymeric adjuncts have been incorporated into hydrogen peroxide gels to mitigate enamel demineralization and limit peroxide diffusion. CaNaTMP has been shown to markedly reduce mineral loss, surface roughness, and peroxide penetration through enamel and dentin, thereby preserving enamel integrity during bleaching [16]. Similarly, bioactive glass, particularly the 45S5 composition, has demonstrated protective benefits when added to peroxide gels, increasing enamel microhardness and maintaining surface morphology without compromising whitening efficacy [26]. Combinations such as chitosan-bioactive glass (CH-BG) have also yielded promising results, enhancing post-bleaching calcium and phosphorus content and achieving remineralization outcomes comparable to commercial MI Paste in vitro [27]. Antioxidants, including quercetin, have further been investigated for their ability to reduce peroxide diffusion and protect enamel, with quercetin-doped hydrogen peroxide gels maintaining enamel integrity while preserving whitening effectiveness [17]. These findings are consistent with systematic reviews on biomimetic mineralization and remineralization strategies [28] and align with broader mechanistic insights into whitening processes [29]. Collectively, such non-polymeric agents may act synergistically with polymer matrices by replenishing ions or repairing enamel surfaces, while polymers primarily regulate gel rheology and release kinetics. Nevertheless, further research is warranted to optimize concentrations, assess compatibility with different gel formulations, and establish long-term clinical outcomes (see Table 1).

### 2.2. Carbamide Peroxide

#### 2.2.1. Whitening Efficacy and Color Evaluation

Carbamide peroxide is widely used in tooth whitening products, both in professional dental treatments and over-the-counter applications [1,7]. Carbamide peroxide acts by gradually decomposing into hydrogen peroxide and urea, allowing a slower and more controlled release of whitening agents compared to the direct application of hydrogen peroxide [3,29]. Meireles et al. [3] evaluated the whitening efficacy of carbamide peroxide in an in vitro study using bovine enamel samples treated with 10%, 16%, and 37% carbamide peroxide concentrations. The 10% and 16% carbamide peroxide were applied for 4 h daily over 14 days, while the 37% concentration was used in three short 20 min sessions with light activation. Although the 37% carbamide peroxide provided faster whitening, all three concentrations resulted in similar overall color changes (ΔE values) after one week, indicating that lower concentrations can achieve comparable results, albeit with a slower onset [3]. Previous studies have indicated that higher concentrations of carbamide peroxide can accelerate whitening effects but may also increase the risks of tooth sensitivity, enamel alterations, and soft tissue irritation [5,6,8]. Conversely, lower concentrations might require longer treatment durations but are generally safer and better tolerated, particularly for unsupervised home whitening [1,7]. Additionally, Krayem et al. [30] conducted a randomized controlled clinical trial comparing two over-the-counter tooth whitening systems, one containing 10% carbamide peroxide. The study reported significant improvements in tooth color with carbamide peroxide use, while also emphasizing the importance of user compliance and the potential for side effects in clinical outcomes [30].

#### 2.2.2. Biocompatibility and Safety Considerations

While carbamide peroxide offers the benefit of a slower release of hydrogen peroxide, concerns persist about its potential cytotoxicity due to its ability to penetrate enamel and dentin and reach the dental pulp. Several studies have explored these biological effects to better clarify its safety profile [6]. Yilmaz and Gul examined the impact of 16% carbamide peroxide on various dental materials, including amalgam, composite resin, and ceramics, and found no significant changes in ion release following repeated carbamide peroxide exposure, suggesting minimal risk of material degradation during home bleaching [31]. However, other studies have indicated that even low concentrations of carbamide peroxide may adversely affect dental pulp cells and related tissues [6]. For example, Fonseca de Lima et al. studied very low carbamide peroxide concentrations (0.0001% to 0.1%) on odontoblast-like MDPC-23 cells and observed morphological alterations, membrane disruption, and decreased cell viability, indicating potential cytotoxic effects even at minimal doses [6]. Collectively, these findings suggest that while carbamide peroxide may be safe for use on dental materials during home bleaching, caution is warranted regarding its biological effects on pulp and pulp-related cells. Consequently, careful regulation of carbamide peroxide concentration, application time, and treatment frequency is crucial to balance whitening efficacy with biocompatibility [6,31]. In addition, incorporation of n-Hap into carbamide peroxide formulations may help reduce enamel demineralization and improve stain-removing efficacy. A recent comprehensive review and an in vitro whitening comparison study support its potential adjunctive role in enhancing enamel appearance [18,19].

#### 2.2.3. Role of Polymer-Based Carriers

Carbamide peroxide whitening products are commonly formulated as gels containing polymers such as Carbopol, which enhance viscosity, improve retention within carrier trays, and enable controlled application during at-home whitening [3,6,25]. Unlike hydrogen peroxide, carbamide peroxide decomposes into hydrogen peroxide and urea, the latter increasing the local pH. A more alkaline environment not only reduces enamel demineralization but also accelerates the decomposition of hydrogen peroxide into water and oxygen radicals, thereby altering its release kinetics and oxidative activity [3,31]. This dual release profile highlights the importance of polymer carriers in maintaining formulation stability and regulating both the peroxide and urea components. Rheological requirements also differ according to application mode. In-office hydrogen peroxide gels require high viscosity to prevent leakage, whereas carbamide peroxide gels designed for tray-based home use rely on moderate viscosity to ensure adequate flow and intimate adaptation to tooth surfaces [1,2,3]. Incorporating protective agents such as CaNaTMP into carbamide peroxide gels has further been shown to mitigate mineral loss and preserve enamel integrity [13]. Because carbamide peroxide decomposes into hydrogen peroxide and urea, its carrier systems share similarities with those used in hydrogen peroxide gels. However, the presence of urea introduces distinct chemical effects, particularly alkalinization, which differentiates its interactions with polymer matrices. Although dedicated studies on polymer–carbamide peroxide interactions remain limited, findings from hydrogen peroxide systems indicate that carriers such as PVP, Carbopol, and HPMC can provide stabilization and diffusion control, effects that are likely relevant for carbamide peroxide formulations as well [9,11,14,20,21]. In particular, the synergy between urea-mediated alkalinization and polymer-controlled release may enhance whitening efficacy while reducing adverse effects during prolonged at-home use [3,6,31]. Therefore, while certain insights can be extrapolated from hydrogen peroxide systems, carbamide peroxide requires separate evaluation to establish polymer-specific effects in its unique dual-release context. Nevertheless, further studies are warranted to clarify the distinct mechanistic roles of polymers in carbamide peroxide stability, release dynamics, and long-term biocompatibility. The main findings on carbamide peroxide efficacy, safety, and delivery systems are summarized in Table 2.

### 2.3. Charcoal Toothpaste

#### 2.3.1. Color Evaluation

The active ingredients in whitening toothpastes are broadly categorized into chemical whitening agents and abrasive particles. Chemical agents such as hydrogen peroxide or carbamide peroxide promote tooth whitening through redox reactions that break down pigmented molecules into smaller, less intensely colored fragments [32]. In contrast, abrasive agents remove extrinsic stains mechanically through friction generated during brushing, aided by the hardness of abrasive particles relative to stains [33,34]. In recent years, activated charcoal has become increasingly popular in oral care products, largely driven by marketing claims emphasizing natural origins and stain-removal capabilities [34,35]. The whitening effect of charcoal toothpastes primarily relies on mechanical abrasion of surface stains and potential adsorption of pigmented molecules. However, these products generally do not significantly affect the intrinsic color of teeth, which is mainly determined by the optical properties of enamel and the underlying dentin [32,34]. Huaman-Sarmiento et al. reviewed the applications of activated charcoal in oral care and noted that although charcoal-containing toothpastes may slightly improve surface brightness due to their abrasive and adsorptive properties, current evidence does not indicate they outperform traditional whitening toothpastes containing silica or low concentrations of peroxide in terms of whitening efficacy [35]. Furthermore, there are concerns regarding the potential for increased enamel wear associated with charcoal formulations [34,35]. Tooth color changes are typically assessed using visual shade guides or more objective instrumental methods, such as spectrophotometers or colorimeters, with instrumental measurements offering greater precision in detecting subtle color differences [34,36].

#### 2.3.2. Biocompatibility and Safety Considerations

Carneiro et al. evaluated charcoal-based toothpastes and demonstrated that these products can cause significantly greater enamel wear and surface irregularities compared to milder whitening formulations, particularly with frequent or prolonged use [37]. Such surface alterations may reduce enamel gloss and increase the risk of extrinsic stain accumulation and plaque retention, potentially diminishing whitening effects and impacting periodontal health [37]. Joiner and Luo reported that excessive abrasiveness in certain whitening toothpastes, including those containing charcoal, may contribute to progressive enamel wear, dentin exposure, increased sensitivity, and compromised tooth structure over time [34]. Huaman-Sarmiento et al. further emphasized that while charcoal’s physical properties may assist in stain removal, clinical evidence supporting its safety and efficacy remains limited, and its potential abrasive effects on enamel require further investigation [35]. Currently, there is no definitive evidence indicating significant cytotoxic effects of charcoal-based toothpastes on oral soft tissues. However, recent in vitro studies reinforce safety concerns. Carneiro et al. demonstrated that activated charcoal toothpastes significantly increase enamel wear compared to conventional formulations, as evidenced by elevated surface roughness and tissue loss [37]. Similarly, Cutrim et al. reported that brushing with charcoal-containing products reduces enamel microhardness and increases surface roughness, although it does not alter tooth color [38]. These findings suggest that frequent use of highly abrasive formulations could increase the risk of enamel surface damage and long-term structural compromise [34]. Although not the primary focus of most studies, some charcoal toothpastes include polymer-based thickening agents such as carbomer or cellulose derivatives, which help increase viscosity and stabilize particles within the formulation [34]. While Huaman-Sarmiento et al. noted the presence of polymers in certain formulations, there is currently insufficient evidence demonstrating significant protective effects of these polymers specifically in charcoal-containing toothpastes [35]. Given these considerations, clinicians should exercise caution when recommending charcoal-based whitening products. Patients should be informed about the limited scientific evidence supporting their efficacy and safety, as well as the potential risks of excessive enamel wear. Furthermore, consumers should also be cautious of marketing claims portraying charcoal toothpastes as “natural,” “chemical-free,” or inherently safer alternatives, as such claims are often not substantiated by robust scientific evidence. The key findings regarding the efficacy, biocompatibility, and formulation considerations of charcoal toothpastes are summarized in Table 3.

### 2.4. Fluoride Compounds

#### 2.4.1. Color Evaluation

Fluoride compounds have been extensively used in dentistry for caries prevention and enamel remineralization, and their inclusion in tooth whitening protocols has attracted considerable interest in recent years. Although fluoride itself does not possess intrinsic bleaching properties, its incorporation into bleaching gels serves to mitigate enamel demineralization and reduce tooth sensitivity, thereby enhancing patient comfort during whitening treatments [32,34,36]. A clinical trial by Mohammadipour et al. [8] assessed the effect of adding 2% sodium fluoride to an in-office hydrogen peroxide bleaching gel. The results indicated that the mean color change (ΔE values) after two weeks was similar between the fluoride-containing and non-fluoride groups (approximately 5.5–5.9; *p* > 0.05). However, patients treated with the fluoride-containing gel reported significantly lower tooth sensitivity scores (1.7 ± 0.8 vs. 3.2 ± 1.0; *p* < 0.001), demonstrating fluoride’s role in reducing bleaching-induced sensitivity. Similarly, El-Damanhoury et al. [39] conducted an in vitro study comparing a calcium silicate–sodium phosphate–fluoride system to a bioactive glass formulation (NovaMin) for enamel remineralization post-bleaching. Their findings showed that the fluoride-containing formulation achieved greater enamel microhardness recovery (94.3% vs. 86.5%) and more effectively minimized mineral loss associated with bleaching [39]. This outcome aligns with fluoride’s established capacity to promote the deposition of calcium and phosphate ions, forming fluorapatite crystals that enhance resistance to acid attacks [34,36]. Although fluoride may not directly increase whitening efficacy, its supportive role in maintaining enamel integrity, alleviating hypersensitivity, and promoting remineralization is essential for the overall success and patient acceptance of bleaching treatments. Recent in vitro studies further support this notion, showing that fluoride-containing formulations, particularly those combined with bioactive glass systems, significantly improve post-bleaching enamel microhardness recovery and reduce mineral loss [40].

#### 2.4.2. Biocompatibility and Safety Considerations

Fluoride is widely recognized for its ability to inhibit demineralization and facilitate remineralization of dental hard tissues. However, its use in bleaching gels warrants careful evaluation regarding both enamel preservation and biological safety [34,36]. Gruba et al. [41] investigated the physicochemical properties of bleaching gels containing 0.5% sodium fluoride and 0.1% nano-sized sodium trimetaphosphate (nano-TPM). Compared to conventional 35% hydrogen peroxide formulations, the fluoride-containing gel significantly reduced enamel surface roughness (Ra values decreased by 25%; *p* < 0.05) and preserved enamel microhardness at approximately 92% of baseline after pH cycling, emphasizing fluoride’s protective influence on enamel surfaces [41]. Moreover, Felipe Akabane et al. [42] evaluated the cytotoxicity of a bleaching gel containing 0.1% sodium fluoride and TPM on human dental pulp cells using a trans-amelodentinal assay. The results demonstrated cell viability exceeding 80% after 72 h of exposure, suggesting favorable biocompatibility [42]. Additionally, the fluoride-containing group exhibited significantly less enamel hardness loss (12% reduction) compared to the traditional formulation (26% reduction; *p* < 0.05), highlighting fluoride’s role in reducing bleaching-induced structural damage. Collectively, these findings indicate that while fluoride does not contribute directly to bleaching action, its incorporation into whitening gels substantially enhances enamel resilience, reduces hypersensitivity, and maintains biocompatibility. Fluoride-containing formulations are particularly beneficial for patients susceptible to post-bleaching sensitivity or enamel demineralization. Although promising, further long-term studies are necessary to determine the optimal concentrations and synergistic combinations of fluoride with other agents for maximizing efficacy and safety in bleaching applications [34]. Although most studies focus on fluoride’s remineralizing and desensitizing properties, polymer-based carriers such as Carbopol or HPMC are frequently used in bleaching gels to improve viscosity, enhance adhesion to tooth surfaces, and modulate the release of active agents. However, specific evidence demonstrating unique benefits of polymer carriers in fluoride-containing whitening formulations remains limited and warrants further investigation [34]. A summary of these key findings is presented in Table 4.

### 2.5. Sodium Bicarbonate

#### 2.5.1. Color Evaluation

Sodium bicarbonate (baking soda) has gained substantial attention as an active ingredient in whitening toothpastes due to its mild abrasivity and stain-removal capacity. Unlike hydrogen peroxide or carbamide peroxide, sodium bicarbonate does not chemically bleach tooth structures but functions primarily through mechanical action that disrupts and removes extrinsic stains from enamel surfaces [1,33]. AlShehri et al. [43] conducted an in vitro study in which enamel specimens were subjected to repeated brushing cycles with various over-the-counter whitening products, including a baking soda-based toothpaste. Their results demonstrated that the baking soda group achieved an average color change of ΔE = 4.2. Although slightly lower than peroxide-based formulations (ΔE = 5.7), this change exceeded the perceptibility threshold of ΔE = 3.3, making it visually appreciable [43]. Similarly, Abidia et al. reported baking soda’s whitening efficacy compared to other natural whitening agents. Using extracted human teeth, they measured both stain removal and enamel surface preservation. Baking soda achieved an average ΔE of 3.8 after seven days of application, outperforming activated charcoal (ΔE = 2.1) and strawberry-based pastes (ΔE = 1.5). Importantly, scanning electron microscopy (SEM) analysis revealed no evidence of enamel erosion or significant surface alterations, emphasizing baking soda’s relatively safe abrasive profile [44]. A systematic review by Li [45] analyzed both in vitro and clinical studies evaluating baking soda-containing dentifrices. The review concluded that toothpastes formulated with 45–65% sodium bicarbonate consistently delivered superior stain removal compared to conventional fluoride toothpaste. One clinical trial noted a 62% reduction in extrinsic stain scores after six weeks of twice-daily brushing with a 65% baking soda toothpaste, compared to only 20–30% stain reduction using standard pastes. These results underscore sodium bicarbonate’s capacity to produce visible esthetic improvements without relying on peroxide-based chemical bleaching agents. Although sodium bicarbonate does not contribute to intrinsic tooth color change through chemical bleaching, existing evidence suggests it offers a valuable balance of moderate stain removal efficacy and enamel safety, making it an appealing alternative for patients seeking non-peroxide whitening options [33,45].

#### 2.5.2. Biocompatibility and Safety Considerations

Sodium bicarbonate’s biocompatibility has been evaluated through both laboratory and clinical studies, which generally confirm its safety when used in appropriate concentrations and professionally formulated products. Mahmiyah et al. [46] assessed the cytotoxicity of various sodium bicarbonate concentrations on human gingival fibroblasts. They found that concentrations ranging from 1% to 7% maintained high cell viability (88–105%), well above the accepted cytotoxic threshold of 50%. However, concentrations of 10% or higher led to significant reductions in cell survival, dropping to approximately 2–3%, underscoring the importance of concentration limits for safe clinical use [46]. Clinical evidence further supports sodium bicarbonate’s safety profile in practical settings. Axe et al. [47] conducted a randomized clinical trial investigating a toothpaste containing 67% sodium bicarbonate combined with sodium hyaluronate, used twice daily for six weeks in patients with mild gingivitis. The study found no adverse effects such as gingival irritation or mucosal injury. Moreover, clinical parameters, including bleeding indices and overall soft tissue health, improved significantly, suggesting that even high-concentration baking soda formulations can be well tolerated when appropriately formulated and used as directed [47]. The discrepancy between in vitro cytotoxicity at higher concentrations and safe clinical use highlights the importance of formulation vehicles, application protocols, and exposure duration in determining biocompatibility. Many baking soda-based formulations also incorporate polymeric binders, such as Carbopol or cellulose derivatives, to enhance paste viscosity, improve particle dispersion, and moderate abrasivity. These polymers help stabilize baking soda particles, potentially reducing localized enamel abrasion during brushing and contributing to a more uniform cleaning effect [1,33]. Nevertheless, specific research evaluating the unique benefits of polymers in baking soda whitening formulations remains limited and warrants further investigation. In summary, sodium bicarbonate demonstrates effective extrinsic stain removal and a favorable safety profile at controlled concentrations. It represents a promising option for patients seeking a gentle, non-peroxide whitening agent, provided its formulation and usage adhere to clinically validated safety parameters [33,45,47]. A summary of these key findings regarding sodium bicarbonate’s whitening efficacy and biocompatibility is presented in Table 5.

### 2.6. Phthalimidoperoxycaproic Acid

#### 2.6.1. Whitening Efficacy and Color Evaluation

Phthalimidoperoxycaproic acid (PAP) is a peracid that does not generate free radicals during its whitening action and has therefore been increasingly investigated as an alternative to conventional hydrogen peroxide and carbamide peroxide. Its mechanism is based on direct oxidation of chromophores within tooth tissues without the formation of ROS. This unique mode of action reduces the potential for oxidative damage to enamel and oral soft tissues and may lower the risk of tooth sensitivity. As a result, PAP represents a theoretically safer whitening agent, especially for patients who are sensitive to peroxide-based products or who prefer peroxide-free options [48]. In vitro studies support the whitening potential of PAP. Pascolutti et al. demonstrated that a PAP+ gel applied in six sessions of 10 min each improved enamel shade by approximately eight VITA Bleachedguide units. This outcome was superior to that obtained with a 6% hydrogen peroxide formulation and, importantly, did not compromise enamel surface integrity [48]. A study conducted by Müller-Heupt et al. further confirmed that PAP effectively removed artificially induced stains. Although the whitening effect was somewhat lower than hydrogen peroxide, PAP treatment did not cause enamel surface alterations under scanning electron microscopy and did not reduce the viability of human fibroblasts. By contrast, hydrogen peroxide produced mild interprismatic dissolution and cytotoxic effects [7]. Clinical data also highlight the promise of PAP. Stübinger et al. reported successful in-office application of a PAP complex that produced noticeable whitening while avoiding the generation of ROS. Enamel surfaces remained intact, as verified by electron microscopy, and patients did not report significant adverse effects [49]. Taken together, these findings indicate that PAP is capable of producing clinically relevant whitening results while reducing the risks associated with peroxide-based treatments.

#### 2.6.2. Biocompatibility and Safety Considerations

PAP has consistently demonstrated a favorable safety profile compared with hydrogen peroxide and carbamide peroxide. Müller-Heupt et al. observed that PAP treatment left enamel surfaces unchanged under SEM evaluation, whereas hydrogen peroxide treatment resulted in mild interprismatic damage [7]. In cell culture models, PAP did not reduce the viability of human fibroblasts, further supporting its biocompatibility. Pascolutti et al. also confirmed that PAP+ gel preserved enamel surface hardness and gloss even after repeated application. This result is in contrast to hydrogen peroxide and carbamide peroxide gels, both of which are known to decrease enamel microhardness after bleaching [48]. The absence of erosion or significant mineral loss indicates that PAP formulations can maintain enamel integrity while delivering effective whitening [7,48]. Stübinger et al. provided additional evidence by showing that PAP application avoids ROS generation and preserves enamel morphology, further substantiating its biocompatibility [49]. Collectively, these data establish PAP as a safer alternative to peroxide-based whitening systems, particularly for individuals who present with preexisting dentin hypersensitivity or enamel fragility.

#### 2.6.3. Formulation Considerations

The effectiveness of PAP is strongly influenced by its formulation. The PAP+ system evaluated by Pascolutti et al. was carefully designed with buffering agents and stabilizers that maintained a near-neutral pH and allowed controlled release of the active peracid. These formulation choices were crucial for minimizing enamel erosion and preserving enamel microhardness while achieving substantial whitening effects [48]. Although specific details regarding polymer-based carriers such as Carbopol or PVP were not emphasized in early reports, there is growing recognition that delivery matrices are essential for the stability and performance of PAP [9,49]. Stübinger et al. described the use of a PAP complex delivered as a composite gel, which provided controlled application and resulted in effective whitening outcomes without enamel alteration [49]. The incorporation of polymers and stabilizing agents in PAP gels is expected to enhance viscosity, improve adhesion to enamel, and prevent premature degradation of the active compound [9,10,11]. Future investigations should aim to clarify how specific polymers and formulation strategies affect PAP stability, release kinetics, and interaction with dental tissues. Well-designed randomized controlled clinical trials of sufficient duration are also required to determine the long-term safety, durability, and patient-reported outcomes of PAP-based whitening products. With continuing advances in formulation technology, PAP has the potential to evolve into a reliable peroxide-free whitening alternative that combines efficacy with superior biocompatibility (see Table 6) [7,49].

### 2.7. Blue Covarine

#### 2.7.1. Color Evaluation

Blue covarine has gained attention as an additive in whitening toothpastes because of its unique optical properties. Instead of chemically bleaching tooth structures, blue covarine deposits fine blue pigments on the enamel surface. This pigment layer modifies how light reflects off the teeth, helping to visually neutralize yellowish tones and create the immediate perception of whiter teeth [36]. However, clinical evidence suggests that the whitening effect of blue covarine remains limited. In a randomized controlled clinical trial conducted by Meireles et al. [50], seventy-five participants were assigned to use either a conventional toothpaste, a blue covarine-containing whitening toothpaste, or a 10% carbamide peroxide bleaching agent over two weeks. The results indicated no significant differences between the blue covarine and conventional toothpaste groups regarding objective tooth color improvement. Parameters such as tooth shade, CIELab values, ΔEab, and ΔE00 showed *p*-values ranging from 0.3 to 0.7. In contrast, the carbamide peroxide group demonstrated significantly greater color changes, with *p* equal to 0.001, and participants in this group also reported higher satisfaction scores. Importantly, both the blue covarine and conventional toothpaste groups exhibited significantly lower rates of tooth sensitivity and gingival irritation compared with the peroxide group, with *p*-values less than 0.01, indicating favorable short-term biocompatibility for blue covarine formulations [50]. In another investigation, Schlafer et al. [51] conducted a triple-blind randomized trial to assess the immediate whitening effect after a single brushing session using a blue covarine-containing silica-based toothpaste. The study found no significant differences between the blue covarine and control groups in the CIEDE2000 ΔE value, where the mean was 1.6 for the blue covarine toothpaste and 1.3 for the control toothpaste. Other parameters such as Whiteness Index (WIO), Whiteness Index Difference (WID), VITA shade measurements, and CIELab values were also not significantly different after one application, with *p*-values above 0.3. Participants’ subjective satisfaction and perception of their tooth color did not significantly differ between the groups, with *p*-values of 0.31 and 0.71, respectively [51]. These observations suggest that although blue covarine may produce a subtle optical masking effect, it has limited capacity to cause meaningful or lasting tooth color changes, especially after a single or short-term use. Taken together, blue covarine-containing toothpastes may offer cosmetic benefits for individuals seeking immediate esthetic improvement. However, they are not a substitute for peroxide-based bleaching agents when intrinsic whitening or long-term color change is desired. Dental professionals should inform patients that the whitening achieved through blue covarine is primarily superficial and optical, and maintaining visible results requires consistent, ongoing use.

#### 2.7.2. Biocompatibility and Safety Considerations

Beyond its whitening effect, blue covarine-containing toothpastes have demonstrated a favorable safety profile in clinical studies. In the randomized clinical trial conducted by Meireles et al. [50], no significant differences were observed in gingival irritation or tooth sensitivity between the blue covarine toothpaste group and the conventional toothpaste group after one and two weeks of use. Moreover, both groups experienced significantly fewer adverse effects compared to the group using a 10% carbamide peroxide bleaching gel, with *p*-values below 0.01, suggesting good short-term biocompatibility for blue covarine-based formulations [50]. Although blue covarine toothpastes are generally regarded as safe, clinicians should advise patients about their limitations. Unlike peroxide-based bleaching agents that penetrate enamel and chemically modify chromophores within tooth structures, blue covarine achieves its effect through surface pigment deposition. This optical effect is temporary and diminishes over time due to factors such as chewing, drinking, and regular oral hygiene practices. Therefore, repeated and consistent use is necessary to maintain the perceived whitening benefit [51]. There is limited research on the long-term effects of continuous blue pigment accumulation on enamel surfaces or on potential interactions between blue covarine and other toothpaste ingredients. Although short-term studies indicate good tolerance, future investigations are necessary to assess whether prolonged use could influence enamel surface properties, roughness, or overall oral health. In addition, while polymer-based carriers are not a primary focus in most studies of blue covarine, they play a supporting role in toothpaste formulations. Ingredients such as Carbopol, HPMC, or other polymers are often used to stabilize pigments like blue covarine within the toothpaste matrix, control viscosity, and improve the even distribution of pigments on the enamel surface. These polymers may help reduce pigment aggregation, ensure consistent color effects, and enhance product handling during use. However, specific studies evaluating the unique benefits of polymers in blue covarine-containing toothpastes remain limited, and further research is warranted to clarify their contribution to both efficacy and safety [1,33,34]. Importantly, in addition to blue covarine, other optical colorants and dye mixtures have also been explored as adjuncts in whitening formulations. Clinical trials involving novel color-correcting serums have demonstrated significant and immediate improvements in perceived tooth whiteness, with no reported safety concerns [52]. Another randomized study assessed bleaching chewing gums containing food-grade dyes (e.g., indigotine [E132] and spirulina), reporting statistically significant improvements in whiteness indices shortly after mastication [53]. These findings suggest that diverse dye systems beyond blue covarine can effectively alter light reflection and enhance tooth brightness in clinical settings. A summary of these key findings regarding blue covarine’s whitening efficacy and biocompatibility is presented in Table 7.

## 3. Conclusions

Tooth whitening remains a fundamental component of esthetic dentistry, driven by the increasing demand for effective, safe, and minimally invasive cosmetic solutions. Among the available agents, hydrogen peroxide and carbamide peroxide exhibit the highest efficacy for intrinsic whitening through oxidative mechanisms. However, their potential to cause enamel damage and cytotoxic effects, particularly at elevated concentrations, underscores the importance of carefully designed clinical protocols and thorough patient education.

Among the alternatives, sodium bicarbonate offers moderate whitening effects mainly through extrinsic stain removal. Fluoride compounds, although lacking intrinsic whitening ability, contribute by reducing demineralization, alleviating hypersensitivity, and preserving enamel integrity. Together, these agents provide valuable and safer options for patients seeking gentler whitening approaches, particularly for those with heightened sensitivity risks. In contrast, charcoal-based products, despite their popularity in consumer markets, lack robust evidence supporting superior whitening efficacy and raise considerable concerns regarding their abrasive effects on enamel. This highlights the critical role of dental professionals in guiding patients toward safe and evidence-based choices. Blue covarine, although capable of delivering immediate optical whitening via pigment deposition, does not induce meaningful intrinsic color change and necessitates continuous use to maintain its cosmetic benefits.

A significant advancement in contemporary whitening formulations is the incorporation of polymer-based delivery systems, including Carbopol, PVP, and HPMC. Beyond improving viscosity and handling, these polymers can stabilize peroxide and influence its release profile. For example, PVP may form hydrogen bonds with hydrogen peroxide, helping to reduce premature decomposition. Such interactions can enhance formulation stability, modulate bleaching efficacy, and improve patient compliance. Clinically, these improvements may contribute to reducing whitening-associated sensitivity, improving patient comfort, and achieving more predictable outcomes. Moreover, the incorporation of protective agents such as CaNaTMP or fluoride within polymer matrices may synergistically preserve enamel microhardness and mitigate adverse effects during whitening procedures.

Overall, modern tooth whitening practices must strike a balance between achieving esthetic results and ensuring patient safety and long-term oral health. Continued research into the molecular interactions between whitening agents and polymer-based carriers, as well as the development of innovative delivery systems, holds significant potential to improve the therapeutic index of these products. Future investigations should focus on elucidating the long-term biocompatibility of emerging agents, clarifying their molecular mechanisms of stabilization and release, and optimizing concentrations and application protocols to achieve desired whitening outcomes while protecting both dental hard and soft tissues. It should also be emphasized that in vitro results, while valuable for mechanistic insights, cannot be directly extrapolated to clinical outcomes, highlighting the necessity of well-designed human studies to confirm both safety and efficacy. A comprehensive overview of these agents and their respective characteristics is provided in Table 8.

## Figures and Tables

**Table 1:** Summary of hydrogen peroxide use in tooth whitening: efficacy, safety, and delivery systems.

Topic	Key Points
Efficacy	■ Hydrogen peroxide is among the most effective tooth-whitening agents [1,2]. ■ Higher concentrations do not always provide superior results; whitening effect depends on protocol and number of applications rather than concentration alone [2,5]. ■ Repeated applications at moderate concentrations can yield outcomes comparable to or better than single high-concentration treatments [2].
Safety	■ High concentrations (e.g., 35%) cause enamel surface irregularities and reduce microhardness (~18.3% reduction reported) [5]. ■ Incorporating CaNaTMP into hydrogen peroxide gels reduces mineral loss, surface roughness, and peroxide diffusion [13]. ■ Thickener and mineral supplementation of bleaching gels influence enamel demineralization [15]. ■ Adjunctive remineralizing agents, including bioactive glass, self-assembling peptides, quercetin, and nano-hydroxyapatite (n-HAp), have shown potential to restore or preserve enamel properties, reduce mineral loss, and improve perceived tooth brightness without compromising whitening efficacy [16,17,18,19].
Carriers	■ Hydrogen peroxide is commonly formulated as gels for controlled placement and reduced soft tissue irritation [2,5]. ■ PVP forms hydrogen bonds with peroxide, stabilizing it against premature decomposition [9,10,20]. ■ Carbopol’s cross-linked polyacrylic acid network creates a viscous hydrogel that acts as a diffusion barrier, limiting deep penetration of peroxide [21,22]. ■ HPMC increases viscosity and modulates release kinetics, enabling gradual oxidative action and reduced sensitivity [11,12]. ■ Advanced polymer-based carriers, combined with protective additives (e.g., calcium, fluoride), improve safety and therapeutic efficacy [9,13,14,15,23,24,25].

**Table 2:** Summary of key findings on carbamide peroxide use in tooth whitening.

Aspect	Key Points
Efficacy	■ Gradual decomposition into hydrogen peroxide and urea allows controlled whitening [3,29]. ■ Lower concentrations (10–16%) effective but slower; higher concentrations (≥37%) act faster but raise sensitivity risk [5,6,8]. ■ OTC clinical trials (10% carbamide peroxide) confirm efficacy, compliance is critical [30].
Safety	■ Safe for restorative dental materials (amalgam, composites, ceramics); no significant ion release [31]. ■ Even very low concentrations (0.0001–0.1%) cause morphological changes and reduce pulp cell viability [6]. ■ Careful regulation of concentration, application time, and frequency essential to balance efficacy and cytotoxicity [6,31]. ■ n-HAp reduces demineralization and enhances stain removal; supported by reviews and in vitro studies [18,19].
Carriers	■ Gels with polymers (e.g., Carbopol) improve viscosity, tray retention, and reduce soft tissue exposure [3,6,25]. ■ Urea by-product increases local pH, reduces demineralization, and alters peroxide kinetics [3,31]. ■ CaNaTMP addition mitigates mineral loss and preserves enamel integrity [13]. ■ PVP stabilizes peroxide via hydrogen bonding, prolonging activity [10,20]. ■ Carbopol cross-linked hydrogel structure acts as diffusion barrier and modulates penetration [21,22]. ■ HPMC regulates viscosity, swelling, and release kinetics for gradual oxidation [11,12,32].

**Table 3:** Summary of the efficacy, safety, and formulation considerations of charcoal toothpastes in tooth whitening.

Aspect	Key Points
Whitening efficacy	■ Acts mainly via abrasion and adsorption of pigmented molecules [34,35,37,38]. ■ Limited or no effect on intrinsic tooth color; generally less effective than peroxide- or silica-based toothpastes [32,34,35]. ■ Tooth color changes are commonly assessed visually or more precisely with instruments (spectrophotometers, colorimeters) [34,36].
Safety	■ Frequent use linked to increased enamel wear, surface roughness, and microhardness reduction [34,35,37,38]. ■ Potential consequences include gloss loss, plaque retention, and higher risk of stain accumulation [34,37]. ■ No conclusive evidence of cytotoxicity on oral soft tissues, but abrasion-related risks remain [34,35,37,38].
Formulation	■ Some charcoal toothpastes contain polymers (e.g., carbomer, cellulose derivatives) to increase viscosity and stabilize particles [34,35]. ■ No strong evidence that polymers provide protective effects against abrasion in charcoal-based formulations [35]. ■ Marketing claims such as “natural,” “chemical-free,” or inherently safer are not substantiated by robust evidence [35].

**Table 4:** Role of fluoride compounds as supportive agents in tooth whitening protocols.

Aspect	Key Points
Whitening efficacy	■ Fluoride itself does not bleach teeth but functions as a supportive agent during bleaching, mainly through remineralization and sensitivity reduction [34,36]. ■ Clinical trial: 2% NaF added to in-office hydrogen peroxide gels showed similar ΔE values (~5.5–5.9) compared to control, but significantly reduced tooth sensitivity (1.7 vs. 3.2; *p* < 0.001) [8]. ■ In vitro study demonstrated higher enamel microhardness recovery with fluoride-containing formulations compared to NovaMin (94.3% vs. 86.5%) [39].
Safety and biocompatibility	■ Fluoride-containing gels enhance enamel microhardness and reduce mineral loss after bleaching [39,40]. ■ Trans-amelodentinal assays showed >80% pulp cell viability after exposure to fluoride-containing gels [42]. ■ Fluoride + nano-TPM formulations reduced enamel hardness loss (12% vs. 26%) compared to conventional gels [41].
Clinical considerations	■ Particularly beneficial for patients prone to sensitivity or enamel demineralization during whitening procedures [8,34,36]. ■ Enhances treatment comfort and supports enamel preservation, improving patient acceptance [39,40,41,42].

**Table 5:** Summary of sodium bicarbonate in tooth whitening.

Aspect	Key Points
Whitening efficacy	■ Removes extrinsic stains through mechanical action, not chemical bleaching [1,33,45]. ■ Achieves perceptible color changes (ΔE ~3.8–4.2), though less than peroxide-based products (ΔE ~5.7) [43,44]. ■ Outperforms some natural agents (e.g., charcoal, strawberry-based pastes) while preserving enamel surface [44]. ■ Systematic review and clinical trials confirm superior stain removal with 45–65% sodium bicarbonate dentifrices (up to 62% stain reduction after 6 weeks) [45].
Safety and biocompatibility	■ Generally safe up to ~7%; higher concentrations (≥10%) reduce cell viability in vitro (to 2–3%) [46]. ■ Clinical studies show high tolerance, no gingival irritation, and improved gingival health with ~67% formulations [47]. ■ Discrepancy between in vitro cytotoxicity and clinical safety underscores importance of formulation, exposure time, and delivery mode [46,47].
Formulation considerations	■ Incorporation of polymers (e.g., Carbopol, cellulose derivatives) improves viscosity, disperses particles, moderates abrasivity, and enhances cleaning uniformity [1,9,33]. ■ Specific experimental evidence on polymer benefits in baking soda pastes remains limited and requires further investigation [9,33].

**Table 6:** Summary of PAP in tooth whitening.

Aspect	Key Points
Whitening efficacy	■ PAP acts through direct oxidation of chromophores without producing ROS [48]. ■ In vitro, PAP+ gel improved tooth shade by ~8 VITA Bleachedguide units, exceeding 6% hydrogen peroxide while maintaining enamel integrity [48]. ■ PAP removes artificially induced stains effectively, although less than hydrogen peroxide [7]. ■ Clinical application of PAP complex produced noticeable whitening with intact enamel surfaces [49].
Safety and biocompatibility	■ PAP does not cause interprismatic enamel dissolution, whereas hydrogen peroxide shows mild surface damage [7]. ■ PAP is non-cytotoxic to human fibroblasts in vitro [7]. ■ PAP+ preserves enamel hardness and gloss after repeated use, unlike hydrogen peroxide and carbamide peroxide [48]. ■ Clinical studies confirmed whitening without enamel erosion or ROS generation [49].
Formulation considerations	■ PAP+ includes buffering agents and stabilizers to maintain near-neutral pH and control peracid release [48]. ■ Composite gel formulations allow controlled application without surface alteration [49]. ■ Polymers such as Carbopol or PVP may enhance viscosity, adhesion, and stability of PAP in whitening gels [9,10,11]. ■ Future research should determine optimal carriers and long-term clinical outcomes [7,49].

**Table 7:** Summary of blue covarine and other optical colorants in tooth whitening.

Aspect	Key Points
Whitening efficacy	■ Blue covarine provides optical whitening by depositing pigments on enamel, without chemical bleaching [36,50,51]. ■ Clinical studies suggest limited objective color change compared to peroxide treatments; effects are temporary and require regular use [50,51]. ■ Other optical colorants and dye mixtures (e.g., color-correcting serums) produce immediate improvements in tooth brightness in clinical trials [52]. ■ Chewing gums containing food-grade dyes (e.g., indigotine [E132], spirulina) significantly improve whiteness indices in clinical studies [53]. ■ Hydroxyapatite and blue covarine toothpastes show comparable whitening potential in vitro [19].
Safety and biocompatibility	■ Generally considered safe short-term, with low sensitivity or irritation [50,51]. ■ Long-term effects of pigment accumulation remain unclear [50,51]. ■ No adverse effects reported in clinical studies using color-correcting serums or dye-containing chewing gums [52,53].
Formulation considerations	■ Polymers (e.g., Carbopol, HPMC, PVP) stabilize pigments, improve viscosity, and aid in uniform distribution [9,10,11,12,14,25]. ■ Evidence for unique polymer benefits in blue covarine and other optical colorant pastes remains limited [1,33,34].

**Table 8:** Summary of tooth whitening agents and polymer-based carriers.

Agent	Whitening Mechanism	Efficacy	Safety and Biocompatibility	Polymer-Based Formulation Role
Hydrogen peroxide	Oxidative bleaching of intrinsic pigments (ROS-mediated)	High intrinsic whitening; protocol optimization often more important than simply raising concentration	High concentrations can reduce microhardness (~18% loss) and cause surface irregularities; cytotoxicity risk increases with dose and exposure	PVP stabilizes peroxide via hydrogen bonding; Carbopol creates a viscous diffusion barrier to limit penetration; HPMC modulates release kinetics; combined with CaNaTMP/fluoride to reduce mineral loss
Carbamide peroxide	Gradual release of hydrogen peroxide and urea	Effective intrinsic whitening; slower onset vs. hydrogen peroxide	Safer for dental materials; potential cytotoxicity to pulp cells even at low doses; sensitivity risk rises with concentration	Carbopol improves tray retention; PVP/HPMC stabilize peroxide and regulate release; urea-induced alkalinization reduces demineralization; additives (e.g., CaNaTMP, n-HAp) provide further protection
Charcoal toothpaste	Abrasive removal of extrinsic stains and adsorption	Limited intrinsic whitening; minor brightness improvement	May increase enamel wear, roughness, and lower microhardness; clinical benefits remain unproven	Polymers (carbomer, cellulose) stabilize particles and improve viscosity, but no strong evidence for protective effects
Fluoride compounds	Supportive remineralization; no intrinsic bleaching	Do not increase ΔE directly but maintain enamel integrity; reduce sensitivity during bleaching	Highly biocompatible; enhance remineralization; reduce enamel hardness loss	Often combined with Carbopol/HPMC to improve viscosity and retention; unique polymer contributions remain unclear
Sodium bicarbonate	Mechanical stain removal (abrasivity)	Moderate extrinsic whitening; ΔE ~3.8–4.2; visible but less than peroxide	Generally safe at ≤7%; high concentrations cytotoxic in vitro; clinically well tolerated	Polymers improve paste viscosity, disperse particles, and may reduce localized abrasivity; specific evidence limited
Blue covarine	Optical whitening via pigment deposition on enamel	Immediate but superficial; no intrinsic whitening; requires repeated use	Safe short-term; long-term pigment accumulation effects unknown	Polymers (PVP/HPMC/Carbopol) stabilize pigments, improve viscosity, and ensure even distribution
PAP	Direct peracid oxidation of chromophores, without ROS generation	Effective whitening; sometimes superior to 6% hydrogen peroxide in vitro; preserves enamel surface	Maintains enamel hardness and gloss; non-cytotoxic to fibroblasts; no interprismatic dissolution observed	Formulated with stabilizers and near-neutral pH; polymers (Carbopol, PVP, HPMC) likely enhance viscosity, adhesion, and stability; further validation needed

## Data Availability

No new data were created or analyzed in this study. Data sharing is not applicable.

## References

[1] Butera A., Maiorani C., Rederiene G., Checchi S., Nardi G.M. (2024). Evaluation of the effectiveness of different types of professional tooth whitening: A systematic review. Bioengineering.

[2] Altınışık H., Nezir M. (2025). Clinical evaluation of in-office bleaching with low, medium, and high concentrate hydrogen peroxide: A 6-month a double-blinded randomized controlled trial. Clin. Oral Investig..

[3] Meireles S.S., Fontes S.T., Coimbra L.A., Della Bona Á., Demarco F.F. (2012). Effectiveness of different carbamide peroxide concentrations used for tooth bleaching: An in vitro study. J. Appl. Oral Sci..

[4] Tomás D.B.M., Pecci-Lloret M.P., Guerrero-Gironés J. (2023). Effectiveness and abrasiveness of activated charcoal as a whitening agent: A systematic review of in vitro studies. Ann. Anat..

[5] Melo M., Fioresta R., Sanz J.L., Pecci-Lloret M.P., Llena C. (2022). Effect of highly concentrated bleaching gels on enamel microhardness and superficial morphology, and the recovery action of four remineralizing agents. BMC Oral Health.

[6] de Lima A.F., Lessa F.C., Gasparoto Mancini M.N., Hebling J., de Souza Costa C.A., Marchi G.M. (2009). Cytotoxic effects of different concentrations of a carbamide peroxide bleaching gel on odontoblast-like cells MDPC-23. J. Biomed. Mater. Res. B Appl. Biomater..

[7] Müller-Heupt L.K., Wiesmann-Imilowski N., Kaya S., Schumann S., Steiger M., Bjelopavlovic M., Deschner J., Al-Nawas B., Lehmann K.M. (2023). Effectiveness and safety of over-the-counter tooth-whitening agents compared to hydrogen peroxide in vitro. Int. J. Mol. Sci..

[8] Mohammadipour H.S., Borouziniat A., Bagheri H., Khorshid M., Shahri A., Shooshtari Z., Rezaei M. (2025). Tooth sensitivity and whitening effect of an in-office bleaching gel containing 2% sodium fluoride: A randomized triple-blind clinical trial. Clin. Oral Investig..

[9] Kida D., Zakrzewska A., Zborowski J., Szulc M., Karolewicz B. (2021). Polymer-based carriers in dental local healing—Review and future challenges. Materials.

[10] Kurakula M., Rao G.S.N.K. (2020). Pharmaceutical assessment of polyvinylpyrrolidone (PVP): As excipient from conventional to controlled delivery systems with a spotlight on COVID-19 inhibition. J. Drug Deliv. Sci. Technol..

[11] Vlad R.A., Pintea A., Pintea C., Rédai E.M., Antonoaea P., Bîrsan M., Ciurba A. (2025). Hydroxypropyl methylcellulose—A key excipient in pharmaceutical drug delivery systems. Pharmaceutics.

[12] Knarr M., Rogers T.L., Petermann O., Adden R. (2025). Investigation and rank-ordering of hydroxypropyl methylcellulose (HPMC) properties impacting controlled release performance. J. Drug Deliv. Sci. Technol..

[13] Fernandes A.V.P., Nunes G.P., Urzedo L.O.R., de Toledo P.T.A., Martins T.P., Alves R.O., Delbem A.C.B. (2025). Total sodium replacement by calcium in trimetaphosphate and its incorporation into dental whitening gels: A novel strategy for in-office treatment. J. Dent..

[14] Yoo D., Ahn J.H., Kang N.G. (2023). Design and characterization of non-erosive polymeric tooth-whitening compositions. Appl. Sci..

[15] Andrade A.C.M., Comba A., Scotti N., Es Sebar L., Torres C.R.G. (2025). Influence of thickener and mineral supplementation of bleaching gels on enamel demineralization. J. Esthet. Restor. Dent..

[16] Elminofy K.M., Hasan M.M.A., Shebl E.A.E. (2024). Evaluation of different remineralizing agents on microhardness and surface roughness of bleached enamel. Tanta Dent. J..

[17] Alves R.O., Nunes G.P., Martins T.P., Toledo P.T.A., Ragghianti M.H.F., Delbem A.C.B. (2025). Effect of quercetin-doped hydrogen peroxide gels on enamel properties: An in vitro study. Gels.

[18] Pushpalatha C., Gayathri V.S., Sowmya S.V., Augustine D., Alamoudi A., Zidane B., Albar N.H.M., Bhandi S. (2023). Nanohydroxyapatite in dentistry: A comprehensive review. Saudi Dent. J..

[19] Enax J., Fandrich P., Schulze zur Wiesche E., Amaechi B.T. (2025). The whitening efficacy of a hydroxyapatite toothpaste and a blue covarine toothpaste: A comparative in vitro study. Dent. J..

[20] Panarin E.F., Kalninsh K.K., Pestov D.V. (2001). Complexation of hydrogen peroxide with polyvinylpyrrolidone: Ab initio calculations. Eur. Polym. J..

[21] Peppas N.A., Bures P., Leobandung W., Ichikawa H. (2000). Hydrogels in pharmaceutical formulations. Eur. J. Pharm. Biopharm..

[22] Suhail M., Wu P.C., Minhas M.U. (2020). Using carbomer-based hydrogels for control of the release rate of diclofenac sodium: Preparation and in vitro evaluation. Pharmaceuticals.

[23] Layek B. (2024). A comprehensive review of xanthan gum-based oral drug delivery systems. Int. J. Mol. Sci..

[24] Burnett C.L., Bergfeld W.F., Belsito D.V., Hill R.A., Klaassen C.D., Liebler D.C., Marks J.G., Shank R.C., Slaga T.J., Snyder P.W. (2011). Final report of the amended safety assessment of PVM/MA copolymer and its related salts and esters as used in cosmetics. Int. J. Toxicol..

[25] Gonçalves I.M.C., Sobral-Souza D.F., Roveda A.C., Aguiar F.H.B., Lima D.A.N.L. (2023). Effect of experimental bleaching gels with polymers Natrosol and Aristoflex on the enamel surface properties. Braz. Dent. J..

[26] Yang S.Y., Han A.R., Kim K.M., Kwon J.S. (2022). Effects of incorporating 45S5 bioactive glass into 30% hydrogen peroxide solution on whitening efficacy and enamel surface properties. Clin. Oral Investig..

[27] Fallahzadeh F., Nouri F., Rashvand E., Heidari S., Najafi F., Soltanian N. (2024). Enamel changes of bleached teeth following application of an experimental combination of chitosan-bioactive glass. BMC Oral Health.

[28] Cao C.Y., Mei M.L., Li Q.L., Lo E.C.M., Chu C.H. (2015). Methods for biomimetic mineralisation of human enamel: A systematic review. Materials.

[29] Kwon S.R., Wertz P.W. (2015). Review of the mechanism of tooth whitening. J. Esthet. Restor. Dent..

[30] Krayem E., Banerjee A., Milly H. (2024). Evaluating the efficiency of two different over-the-counter tooth whitening systems: A randomised controlled clinical trial. BDJ Open.

[31] Yilmaz M.N., Gul P. (2024). Effect of carbamide peroxide treatment on the ion release of different dental restorative materials. BMC Oral Health.

[32] Carey C.M. (2014). Tooth whitening: What we now know. J. Evid. Based Dent. Pract..

[33] Joiner A. (2010). Whitening toothpastes: A review of the literature. J. Dent..

[34] Joiner A., Luo W. (2017). Tooth colour and whiteness: A review. J. Dent..

[35] Huaman-Sarmiento E., Mayta-Tovalino F., Munive-Degregori A.A., Mendoza R., Barja-Ore J., Mauricio-Vilchez C. (2023). Uses and applications of activated charcoal in the manufacture of toothpastes and oral rinses: A narrative review. J. Int. Oral Health.

[36] Joiner A. (2004). Tooth colour: A review of the literature. J. Dent..

[37] Carneiro B.T., Kury M., Lopes J.C., Gonçalves R.S., Suzuki T.Y.U., Picolo M.Z.D., Giannini M., André C.B. (2023). Effect of whitening toothpastes and activated charcoal powder on enamel wear and surface roughness. Braz. Oral Res..

[38] Cutrim E.A.C., Penha K.J.S., Silva P.B., Carvalho E.M., Silva M.G., Kugelmeier C.L., Firoozmand L.M. (2024). Optical, mechanical, and chemical impact of brushing with activated charcoal toothpowder and toothpaste on dental enamel: An in vitro evaluation. Materials.

[39] El-Damanhoury H.M., Elsahn N.A., Sheela S., Bastaty T. (2021). In vitro enamel remineralization efficacy of calcium silicate-sodium phosphate-fluoride salts versus NovaMin bioactive glass, following tooth whitening. Eur. J. Dent..

[40] Ergucu Z., Yoruk I., Erdoğan A., Boyacıoğlu H., Hill R., Baysan A. (2023). The use of toothpastes containing different formulations of fluoride and bioglass on bleached enamel. Materials.

[41] Gruba A.S., Nunes G.P., Marques M.T., Danelon M., Alves R.O., de Toledo P.T.A., Briso A.L.F., Delbem A.C.B. (2023). Influence of bleaching gels formulated with nano-sized sodium trimetaphosphate and fluoride on the physicochemical, mechanical, and morphological properties of dental enamel. J. Dent..

[42] Felipe Akabane S.T., Danelon M., Nunes G.P., Gruba A.S., Alberto de Souza-Costa C., Caroline de Oliveira Duque C., de Oliveira Gallinari M., Fraga Briso A.L., Botazzo Delbem A.C. (2021). Evaluation of the aesthetic effect, enamel microhardness and trans-amelodentinal cytotoxicity of a new bleaching agent for professional use containing trimetaphosphate and fluoride. J. Mech. Behav. Biomed. Mater..

[43] AlShehri A., AlRefeai M.H., AlZamil F., AlOtaibi N., AlKinani Y. (2022). Effect of over-the-counter tooth-whitening products on enamel surface roughness and microhardness. Appl. Sci..

[44] Abidia R.F., El-Hejazi A.A., Azam A., Al-Qhatani S., Al-Mugbel K., AlSulami M., Khan A.S. (2023). In vitro comparison of natural tooth-whitening remedies and professional tooth-whitening systems. Saudi Dent. J..

[45] Li Y. (2017). Stain removal and whitening by baking soda dentifrice: A review of literature. J. Am. Dent. Assoc..

[46] Mahmiyah E., Susatyo J.H., Ningsih N.S. (2023). Cytotoxicity of sodium bicarbonate solution to human gingival fibroblast cells. J. Inf. Kesehat..

[47] Axe A., Patel N., Qaqish J., Ling M.R., Araga M., Parkinson C., Goyal C.R. (2024). Efficacy of an experimental toothpaste containing sodium bicarbonate, sodium hyaluronate and sodium fluoride on gingivitis. BMC Oral Health.

[48] Pascolutti M., de Oliveira D. (2021). A radical-free approach to teeth whitening. Dent. J..

[49] Stübinger S., Altenried S., Ren Q. (2024). Tooth-whitening with a novel phthalimido peroxy caproic acid: Short communication. Clin. Cosmet. Investig. Dent..

[50] Meireles S.S., de Sousa J.P., Lins R.B.E., Sampaio F.C. (2021). Efficacy of whitening toothpaste containing blue covarine: A double-blind controlled randomized clinical trial. J. Esthet. Restor. Dent..

[51] Schlafer S., Poulsen P.N., Johansen J., Trap L., Leite F.R.M. (2021). The whitening effect of single brushing with blue-covarine containing toothpaste-A randomized controlled trial. J. Dent..

[52] Pascolutti M., Tomic A., Milleman K.R., Milleman J.L., Walsh L.J. (2024). Safety and effectiveness of a novel color corrector serum for causing temporary changes to tooth shade: A randomized controlled clinical study. Dent. J..

[53] Porciani P.F., Perra C., Grandini S. (2022). Whitening optical effect of new chewing gums. Open Dent. J..

